# Evidence of apoptosis as an early event leading to cyclophosphamide-induced primordial follicle depletion in a prepubertal mouse model

**DOI:** 10.3389/fendo.2024.1322592

**Published:** 2024-10-14

**Authors:** Xia Hao, Arturo Reyes Palomares, Amandine Anastácio, Kui Liu, Kenny A. Rodriguez-Wallberg

**Affiliations:** ^1^ Department of Oncology and Pathology, Karolinska Institutet, Stockholm, Sweden; ^2^ Laboratory of Translational Fertility Preservation, BioClinicum, Stockholm, Sweden; ^3^ Shenzhen Key Laboratory of Fertility Regulation, Center of Assisted Reproduction and Embryology, The University of Hong Kong-Shenzhen Hospital, Shenzhen, China; ^4^ Department of Obstetrics and Gynecology, Li Ka Shing Faculty of Medicine, The University of Hong Kong, Hong Kong, Hong Kong SAR, China; ^5^ Department of Reproductive Medicine, Division of Gynecology and Reproduction, Karolinska University Hospital, Stockholm, Sweden

**Keywords:** ovary, primordial follicle, growing follicle, cyclophosphamide, apoptosis, overactivation, gene expression, timepoint

## Abstract

**Introduction:**

The mechanisms leading to ovarian primordial follicle depletion following gonadotoxic chemotherapy with cyclophosphamide and other cytotoxic drugs are currently understood through two main explanatory theories: apoptosis and over-activation. Discrepancies between the findings of different studies investigating these mechanisms do not allow to reach a firm conclusion. The heterogeneity of cell types in ovaries and their different degrees of sensitivity to damage, cell-cell interactions, periodical follicle profile differences, model age-dependent differences, and differences of exposure durations of tested drugs may partially explain the discrepancies among studies.

**Methods:**

This study used intact prepubertal mice ovaries in culture as study model, in which most follicles are primordial follicles. Histological and transcriptional analyses of ovaries exposed to the active metabolite of cyclophosphamide 4-hydroperoxycyclophosphamide (4-HC) were carried out via a time-course experiment at 8, 24, 48, and 72 h.

**Results:**

4-HC treated ovaries showed a significant decrease in primordial follicle density at 24 h, along with active DNA damage (TUNEL) and overexpressed apoptosis signals (cleaved-poly ADP ribose polymerase in immunohistochemistry and western blotting). Meanwhile 4-HC treatment significantly up-regulated *H2ax, Casp 6, Casp 8, Noxa,* and *Bax* in ovaries, and up-regulated *Puma* in primordial follicles (FISH).

**Discussion:**

Our results indicated that cyclophosphamide-induced acute ovarian primordial follicle depletion was mainly related to apoptotic pathways. No evidence of follicle activation was found, neither through changes in the expression of related genes to follicle activation nor in the density of growing follicles. Further validation at protein level in 4-HC-treated prepubertal mice ovaries at 24 h confirmed these observations.

## 1 Introduction

Women are born with a fixed number of ovarian primordial follicles, which are endowed from their fetal stages, and this number progressively decreases throughout the reproductive lifespan. These dormant primordial follicles constitute the ovarian follicle pool. From puberty onset, cohorts of primordial follicles are periodically activated to grow and develop through different stages, accompanied by the growth of oocytes, the proliferation of surrounding granulosa cells, and the differentiation of some granulosa cells. Among these activated primordial follicles, only a minority continuously develop until final ovulation, whereas the majority will undergo atresia at various stages. The reproductive lifespan of women is biologically determined by the size of their primordial follicle pool and the rates of their activation (1, 2). The functions of several proteins have been associated with the maintenance of primordial follicle quiescence and also their survival during dormancy. These proteins include phosphatase and tensin homolog deleted on chromosome 10 (PTEN), tuberous sclerosis complex (TSC), forkhead box O3 (FOXO3a), cyclin-dependent kinase inhibitor 1B (p27^Kip1^), forkhead box L2 (FOXL2) and anti-Müllerian hormone (AMH), phosphatidylinositol 3 kinase (PI3K), 3-phosphoinositide dependent kinase 1 (PDK1), mammalian target of rapamycin complex 1 (mTORC1) and ribosomal protein S6 (RPS6) (3). Meanwhile, several pathways have been recognized in mediating primordial follicle activation, including the PI3K/PTEN/protein kinase B (Akt) pathway, tuberous sclerosis complex (TSC)-mTORC1-RPS6 signaling pathway, Hippo pathway, mitogen-activated protein kinase (MAPK3/1, also known as extracellular signal-regulated kinase (ERK) 1/2) pathway, and the c-Jun-N-terminal kinase (JNK) pathway (3–6). Additional complexity in studying these mechanisms arises in the interaction between the pathways, which may also involve new pathways and influence multiple functions in the ovaries. Exposure to factors that influence these proteins and pathways resulting in the over-activation or death of primordial follicles will accelerate the reduction of the ovarian follicle pool. This may cause an early onset of menopause and lead to the development of premature ovarian insufficiency (POI) in women.

Alkylating agents including cyclophosphamide (CPA) are commonly used in chemotherapy protocols for cancer and also for treatment of severe benign diseases. CPA has been demonstrated to have a gonadotoxic impact, affecting female fertility (7, 8). After administration, CPA is metabolically converted by the cytochrome P450 system into 4-hydroxycyclophosphalmide (4-OHC), which rapidly interconverts to aldophosphamide. Aldophosphamide spontaneously fragmentates into phosphoramide mustard and acrolein. 4-hydroperoxycyclophosphamide (4-HC) can generate 4-OHC through a non-enzymatic reaction, thus 4-HC is widely used in *in vitro* studies on CPA (9). The CPA active metabolites form DNA adducts or induce DNA double-strand breaks, increasing the generation of γ-H2AX (10). This damage prevents DNA replication and further protein synthesis, thus causing cell death (11).

Several experimental models including rodents, non-human primates, rodents bearing human ovarian tissue grafts, and *in vitro* cultured ovarian tissue, follicle, and cell studies have been used to investigate the ovarian toxicities of chemotherapy. Several mechanisms have been proposed to explain this phenomenon, including 1) oxidative stress, which results in an imbalance between reactive oxygen species (ROS) and cellular endogenous antioxidant superoxide dismutase (SOD), thereby damaging the intra-ovarian environment (12, 13); 2) apoptosis, as a consequence of unrepaired DNA damage and uncorrected cellular stress (14–26); 3) primordial follicle over-activation mediated by the PI3K/PTEN/Akt pathway and the Hippo pathway, leading to premature depletion of the ovarian reserve (9, 25, 27–35); and 4) damage to the ovarian micro-vessel network, resulting in compromised vascularization and increased fibrosis, which indirectly induces primordial follicle loss (15, 36–39). However, a firm conclusion has not yet been reached. Divergences between studies could be related to diverse experimental approaches, the heterogeneities of ovarian cells and their sensitivities to external damages, age-dependent differences, the duration of exposure, or the time frame after exposure. This last aspect, frequently disregarded, is relevant to the experimental design and critical for capturing early or late key biological processes that lead to primordial follicle depletion under specific interventions.

In this study, we provide a comprehensive overview of the potential mechanisms associated with the early changes induced by CPA within a time frame of 72 h that lead to acute depletion of ovarian primordial follicles and ovarian damage. Prepubertal mice ovaries were chosen due to their richness in primordial follicles, increasing the chance to capture effects in the primordial follicle pool. Ovaries were cultured *in vitro* after a single exposure to 4-HC in a time-course experiment and examined at 8, 24, 48, and 72 h. Histological profiles (H&E staining, YBOX2, TUNEL, cleaved-poly ADP ribose polymerase (PARP), and P21) and gene expression analyses of genes related to apoptosis, primordial follicle activation, follicular development, and angiogenesis were investigated. After determining the time point at which significant ovarian primordial follicle depletion occurred, we performed visualizations of *Pten* and *Puma* and quantifications of key proteins related to apoptosis and follicle activation processes at that time point.

## 2 Materials and methods

The chemicals and materials used in this study were purchased from Sigma–Aldrich^®^, Thermo Fisher Scientific^®^, or Gibco ^TM^, if not otherwise indicated at their first appearance in the article.

### 2.1 Animals and ovary acquirement

Ovaries from postnatal day (PND) 4 (n=32) and PND 7-9 (n=9) inhouse-bred B6CBA/F1 female mice were collected in Leibovitz 15 medium enriched with 10% fetal bovine serum (FBS), 100 IU/mL penicillin, and 100 µg/mL streptomycin. The surrounding tissue was removed from the ovaries using Micro-Fine U-100 insulin syringes (BD Medical, USA) under a stereomicroscope (SMZ800N Nikon^®^, France). All procedures involving mice were conducted in accordance with accepted standards of humane animal care and were approved by the Karolinska Institutet and the regional ethics committee for animal research, identified as Dnr 1372 (Date 2018-01-24).

### 2.2 Grouping and cyclophosphamide *in vitro* treatment

Intact left and right ovaries from each mouse were randomly allocated into either the 4-HC-treated group or the control group. Ovaries were placed inside a Millicell^®^ cell culture insert with even distribution and no more than five ovaries were put inside a single insert. The ovarian tissue culture medium was α-Minimal Essential Medium GlutaMAX enriched with 10% FBS, 25 µg/mL transferrin, 5 IU/mL penicillin, and 5 µg/mL streptomycin. Culture medium was added into the wells of a 24-well plate and an insert was carefully placed on top of the medium (9), avoiding the formation of bubbles between the contact surfaces. Before use, the aforementioned prepared culture plate with inserts were pre-equilibrated in humidified conditions at 37°C and 5% CO_2_. In the 4-HC-treated wells, 4-HC (Santa Cruz, USA) dissolved in anhydrous tetrahydrofuran (THF) and dimethyl sulfoxide (DMSO) was added into the culture medium to obtain a final concentration of 5 µM. The final amounts of THF and DMSO were both 0.03%, v/v. The dose of 4-HC was set at 5 µM, based on previous research that indicated that this is within the concentration range at which 4-HC damages both primordial and small primary follicles (21). In the control wells, equal amounts of solvents were added to the culture medium. After 48 h of culturing, half of the culture medium in each well was refreshed without refreshment of 4-HC or the solvents. PND4 mouse ovaries from both groups were collected at 8, 24, 48, and 72 h of culturing for histological (n=3-5/group) and gene expressional analyses (n=3-5/group). PND7-9 mouse ovaries from both groups (n=9/group) were collected at 24 h for Western blot analysis.

### 2.3 Follicle density determination

Ovaries were collected in tubes with 4% formaldehyde (VWR, Sweden), fixed overnight at 4°C, transferred into 70% ethanol, and individually embedded in paraffin. Ovaries were serially sectioned at 5 µm thickness and every 10^th^ section was used for follicle density determination. Other sections were kept for immunohistochemistry staining and fluorescence *in situ* hybridization (FISH). For follicle density determination, selected tissue section loading slides were deparaffinized in xylene followed by rehydration in an ethanol series. The slides were then stained with hematoxylin followed by eosin (H&E staining). Finally, slides were sequentially immersed in ethanol series and xylene and then mounted with PERTEX mounting medium (Histolab, Sweden). Stained slides were scanned using a digital slide scanner (3Dhistech^®^, Hungary) and viewed using Pannoramic Viewer software (3Dhistech^®^, Hungary). Follicle density determination was performed by two independent observers blinded to grouping information. Primordial follicles were counted when only a few flattened squamous granulosa cells surrounded the oocyte and growing follicles (including transitory, primary, secondary follicle, and other pre-antral follicles) were counted when at least one surrounding granulosa cell was cuboidal. Only follicles with a visible and non-pyknotic nucleus in the oocyte were counted. The area (mm^2^) of each of the observed sections was obtained using the measurement tool in the Pannoramic Viewer software. Primordial and growing follicle densities were calculated as the total number of primordial and growing follicles divided by the total area of the observed tissue sections (40).

### 2.4 Immunohistochemistry staining

Tissue section loading slides were deparaffinized in xylene which was followed by rehydration in an ethanol series. The slides were then heated in a Diva Decloaker (Biocare Medical, Sweden) for 20 min for antigen retrieval. Endogenous peroxidase activity was inhibited by Ultra V hydrogen peroxide block. Ultra V block was applied to sections to avoid non-specific bindings. Primary antibodies against YBOX2 (Abcam, 33164, 1:500), p21 (Abcam, 188224, 1:2000) and cleaved-PARP (Cell Signaling, 94885, 1:200) were incubated with slides for hybridization in a humidified chamber at 4°C overnight. Mouse liver tissue sections were used as negative controls. Primary antibody amplifier quanto and HRP polymer quanto were applied in turn. Signals were revealed with a diaminobenzidine (DAB) quanto chromogen which was followed by counterstaining with hematoxylin. The slides were then sequentially immersed in ethanol series and xylene, and mounted with PERTEX mounting medium.

To assess DNA fragmentation after the 4-HC treatment, a terminal deoxynucleotidyl transferase dUTP nick end labeling (TUNEL) assay kit-HRP-DAB (Abcam, ab206386) was used. Briefly, deparaffinized section-loaded slides were permeabilized by heating in Diva Decloaker. After incubation with TdT equilibration buffer, the labeling reaction mixture was prepared and applied to the sections. Following treatment with stop buffer and blocking buffer, conjugate and DAB solutions were applied. Slides were subsequently immersed in ethanol series and xylene, and mounted with PERTEX mounting medium. All sections were counterstained with hematoxylin. Positive control sections were treated with 2.73 Kunitz units/µL Dnase I (QIAGEN, Germany) instead of TdT equilibration buffer. Negative control slides were treated using ddH_2_O instead of the labeling reaction mixture.

Both experiments were performed on a minimum of three biological replicates. Stained slides were scanned using a 3Dhistech^®^ digital slide scanner and viewed using the Pannoramic Viewer software, and the captured images were qualitatively analyzed.

### 2.5 Masson’s staining

Slides were processed for Masson’s staining using the Trichrome Stains kit according to the manufacturer’s protocol. Briefly, after deparaffinization and rehydration, the slides were mordanted in pre-heated Bouin’s Solution at 56°C for 15 min. After rinsing, the slides were stained in working Weigert’s iron hematoxylin solution for 5 min and then rinsed again. The slides were then stained in Biebrich scarlet-acid fuchsin for 5 min, and after being rinsed once again, the slides were placed in working phosphotungstic/phosphomolybdic acid solution for 5 min followed by Aniline blue solution for 5 min and 1% acetic acid for 2 min. After a final rinse and dehydration, the slides were mounted with PERTEX mounting medium. Three biological replicates were used and a section of human lung tissue was used as positive control. Stained slides were scanned by 3Dhistech^®^ digital slide scanner and viewed using the Pannoramic Viewer software. The captured images were qualitatively analyzed.

### 2.6 Fluorescence *in situ* hybridization

RNAscope fluorescent multiplex assay was performed to locate expression of p53 upregulated modulator of apoptosis (*Puma*) and *Pten* (primordial follicle dormancy maintenance related) at the timepoint of 24 h. The assay was performed according to the manufacturer’s instructions, Advanced Cell Diagnostics (ACDBio, USA) and using an automatic stainer (Leica Biosystems BOND-RxM, Triolab AB, Sweden). A bacterial enzyme (DAP-B) was used as a negative control. Three separate probes tested mRNA quality/degradation in samples and reading interferences between different channels. These probes targeted housekeeping genes; *Pol-r2a* (C1 channel), *PpiB* (C2 channel) and *Ubc* (C3 channel). Sections were counterstained with RNAscope DAPI (ACDBio, USA) and mounted with mounting medium (VECTASHIELD^®^, CA, USA). Fluorescent images were acquired with Nikon ECLIPSE Ti Series microscope combined with Nikon’s NIS-Elements imaging software. The experiment was performed on a minimum of three biological replicates.

### 2.7 Quantitative reverse transcription polymerase chain reaction

Total RNA from individual ovaries was extracted from collected ovaries with mechanical homogenization (TissueLyser LT, QIAGEN, Germany) using the Rneasy Micro Kit (QIAGEN, Germany) and cDNA synthesis was performed with the High-Capacity cDNA Reverse Transcription Kit according to the manufacturers’ instructions.

Quantitative reverse transcription polymerase chain reaction (RT-qPCR) analysis was performed in triplicates with the SsoAdvanced Universal SYBR Green Supermix (BIO-RAD, USA) on the QuantStudio 7 Flex RT-qPCR system. The oligonucleotide primers of the target genes were either self-designed or were primers used in other papers (41–43). Primer selection was carried out using a primer designing tool from National Center for Biotechnology Information ( https://www.ncbi.nlm.nih.gov/tools/primer-blast/), with the following setting criteria: PCR product size min of 70 and max of 200 bp; primer melting temperatures (Tm): min 57.0, opt 59.0 or 60.0, max 62.0, and a max Tm difference of 1 or 2; primer must span an exon-exon junction. The search mode was set to automatic and the database was set to Refseq mRNA under the Organism section of Mus musculus (taxid:10090). All other unspecified settings were kept as default. *β-actin (Actb)* was used as an internal control for normalization. The used primer sequences are listed in **Table 1** and the primers were produced by Integrated DNA Technologies (IDT, Sweden). The following cycler regime was used for RT-qPCR reactions: 30 s at 95°C, 40 cycles of 15 s at 95°C, 30 s at 60°C, and a melt curve stage of 5 s at 65°C and 5 s at 95°C. Only data resulting from reactions producing uni-peak melt curves were used for further analysis. After normalization using the corresponding levels of the housekeeping gene *β-actin*, the ΔΔCt fold change in the mRNA levels was calculated relative to the untreated control samples (44), and the relative expressional levels of each gene in the treated and control groups were compared using GraphPad Prism (Version 9.4.1).

**Table 1:** Oligonucleotide primer sequences used for the RT-qPCR analysis.

Gene	Primer sequence (5’-3’)	Product length (bp)	Accession number
*Bax*	F: ATCCAAGACCAGGGTGGCTG	148	NM_007527.3
	R: CAGTCCAAGGCAGTGGGAG		
*H2ax*	F: GTGCTGCTGCCCAAGAAGA	138	NM_010436.2
	R: CCTTTGTGGAGGTGTGGGGA		
*Pten*	F: ACTTTGAGTTCCCTCAGCCA	93	NM_008960.2
	R: ACATTTTGTCCTTTTTGAGCATCT		
*Tap63 (Trp63)*	F: CTGCTCATCATGCCTGGACT	124	NM_001127264.1
	R: CCAGATGGCATGTCGGAACT		
*Ybx2*	F: CCCAACAGCCTACCACAGAA	173	NM_016875.3
	R: GATAGGCGCGGAGGTCTCT		
*Noxa (Pmaip1)*	F: GAGTGCACCGGACATAACTG	107	NM_021451.2
	R: CGTCCTTCAAGTCTGCTGG		
*Puma (Bbc3)*	F: TTGAACAGTGAACACTCTTTTTGT	70	NM_133234.2
	R: CATGTCCTTACAGGTAGTGCC		
*Casp 3*	F: GAGCTTGGAACGGTACGCTAA	114	NM_001284409.1
	R: CCACTGACTTGCTCCCATGT		
*Casp 6*	F: GCTCAAAATTCACGAGGTG	110	NM_009811.4
	R: TTTTGGCGTCGTATGCGTAA		
*Casp 8*	F: ATCGGTAGCAAACCTCTGTGA	169	NM_009812.2
	R: GGTAGAAGAGCTGTAACCTGTG		
*Pdk1*	F: GATCCTGTCACCAGCCAAAA	104	NM_001360002.1
	R: CCACCGAACAATAAGGAGTGC		
*Pcna*	F: TTGCACGTATATGCCGAGACC	104	NM_011045.2
	R: CCGCCTCCTCTTCTTTATCCA		
*Mki67*	F: TTTAACTCAGCGCCTTGTTCC	167	NM_001081117.2
	R: GCATTCCCTCACTCTTGTTAATG		
*P21 (Cdkn1a)*	F: ATCCAGACATTCAGAGCCACA	181	NM_007669.5
	R: AGTCAAAGTTCCACCGTTCTC		
*Sod1*	F: GCGGATGAAGAGAGGCATGTT	121	NM_011434.2
	R: GGCCAATGATGGAATGCTCT		
*Trp53*	F: CCGAAGACTGGATGACTGC	167	NM_001127233.1
	R: CTCCTCAACATCCTGGGGC		
*Bcl-2*	F: TCTTTGAGTTCGGTGGGGTC	154	NM_009741.5
	R: AGTTCCACAAAGGCATCCCAG		
*Foxo3a*	F: CTCATGGATGCTGACGGGTT	174	NM_001376967.1
	R: GTTTGAGGGTCTGCTTTGCC		
*Mtor*	F: GTTGGCATAACAGATCCTGACCC	125	NM_020009.2
	R: TGGTCATTCAGAGCCACAAAC		
*Amh*	F: TTGCTGAAGTTCCAAGAGCC	137	NM_007445.3
	R: GTAGGGCAGAGGTTCTGTGTG		
*Tgfb1*	F: AACTATTGCTTCAGCTCCACAG	159	NM_011577.2
	R: CTGTGTGTCCAGGCTCCAAA		
*Smad2*	F: TTGCCTCCAGTCTTAGTGCC	149	NM_001252481.1
	R: CCAGGTGGTGGTGTTTCTGG		
*Smad3*	F: TCAGAGAGTAGAGACGCCAGTT	167	NM_016769.4
	R: CAGGAGGTGGGGTTTCTGGAATA		
*Stat3*	F: GGAAGCGAGTGCAGGATCTA	97	NM_213659.3
	R: TCTCCTTGGCTCTTGAGGGT		
*Yap1*	F: TCCAACCAGCAGCAGCAAAT	(41)	NM_001171147
	R: TTCCGTATTGCCTGCCTGCCGAAA		
*Mst1*	F: CAGCCCGAGGAAGTGTTTGA R: CACGGGCACTTGCTTGATTG	(41)	XM_006499963.1
*Lats2*	F: CTTTGCTTCCCTCGGACAGT	(41)	NM_010690.1
	R: AACTGGGTGTAGTCAGGGGT		
*Kitl*	F: TCAACTATGTCGCCGGGATG R: CGAAATGAGAGCCGGCAATG	(42)	(42)
*Cd31*	F: CCAAAGCCAGTAGCATCATGGTC R: GGATGGTGAAGTTGGCTACAGG	(43)	NM_008816
*β-actin (Actb)*	F: TCCTTCTTGGGTATGGAATCCT	81	NM_007393.5
	R: TTTACGGATGTCAACGTCACAC		

### 2.8 Western blotting

Ovaries (n=3 per group, in triplicate) were homogenized with a mortar and pestle and cooled by liquid nitrogen; total proteins were extracted with RIPA buffer containing Halt^™^ Protease and Phosphatase Inhibitor Cocktail. After heating the equivalent amount of samples in NuPAGE LDS sample buffer (Invitrogen, Germany) and dithiothreitol solution, the samples and PageRuler Plus Prestained Protein Ladder (10-250 kDa) were separated in NuPAGE 4-12% Bis-Tris gel (Invitrogen, Germany) by electrophoresis with NuPAGE MES SDS running buffer (Invitrogen, Germany) and were then transferred to nitrocellulose membranes using the Trans-blot Turbo RTA Mini Nitrocellulose Transfer kit (Bio-Rad, USA).

The membranes were incubated with 10% nonfat milk for 1 h at room temperature to block nonspecific binding followed by incubation with corresponding primary antibodies overnight at 4°C: phospho-S6 ribosomal protein (Ser235/236) (Cell Signaling, 4858, 1:2000), S6 ribosomal protein (5G10) (Cell Signaling, 2217, 1:1000), FOXO3a (D19A7) (Cell Signaling, 12829, 1:1000), phospho-FOXO3a (S253) (Abcam, 154786, 1:5000), Akt (Cell Signaling, 4858, 1:1000), phospho-Akt (Thr308) (C31E5E) (Cell Signaling, 2965, 1:1000), and cleaved-PARP (Cell Signaling, 94885, 1:1000), with GAPDH (Santa Cruz, 32233, 1:5000) used as an internal control.

After rinsing with tris-buffered saline containing 0.1% Tween 20 detergent (TBST), the membranes were incubated with anti-rabbit IgG (DAKO, P0448, 1:2000) or anti-mouse IgG secondary antibodies (GE Healthcare, NA931V, 1:5000) for 1 h at RT. After rinsing with TBST, the membranes were visualized using Clarity Western ECL Substrate (Bio-Rad, USA) and imaged using the iBright analysis system. The band densities were quantified using ImageJ software (Fiji, National Institutes of Health, USA). The obtained band densities of the target proteins were normalized to the corresponding GAPDH band densities of each sample.

### 2.9 Statistical analysis

The follicle densities, ΔΔCt fold changes, and relative protein levels between the control and 4-HC-treated groups at each time point were compared by unpaired t-tests using GraphPad Prism (Version 9.4.1). Differences were considered statistically significant at *p* < 0.05, and highly significant at *p* < 0.01, two-sided (45).

## 3 Results

### 3.1 Histological analysis of ovarian profile changes induced by CPA

**Figure 1:**
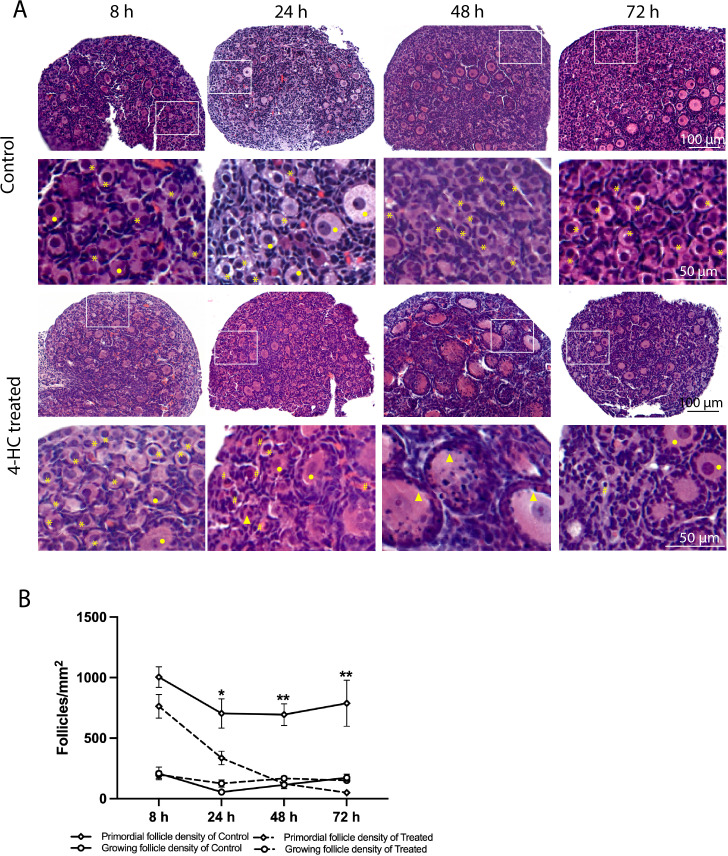
**(A)** H&E-staining in control and 4-HC-treated ovaries over time. Each one involves an overview of the ovary and magnification of the cortex area marked by a square in the overview above. Marks: primordial follicles (*), growing follicles (circle), pyknotic nuclei in oocytes (#), growing follicles with some granulosa cells showing pyknotic nuclei (triangles). **(B)** Primordial follicle density and growing follicle density in the control and 4-HC-treated groups over time. Follicle density was expressed as follicle number/mm^2^, mean with SEM. Comparing with control group: ^*^ *p* < 0.05, ^**^ *p* < 0.01.

A dynamic progression of morphological changes in the ovaries treated with 4-HC was evidenced in the histological assessment within the 72-h follow-up period (**Figure 1A**). At the earliest observation at 8 h, the 4-HC-treated ovaries did not substantially differ compared to controls. However, at 24 h, pyknotic nuclei were observed in the oocytes of the primordial follicles of the 4-HC-treated ovaries, suggestive of direct damage to the immature oocytes induced by CPA. Primordial follicle depletion was evident at 24 h and it had progressed at 48 h with significantly fewer primordial follicles in the cortex area and an increase in pyknotic nuclei in the granulosa cells of the growing follicles. At 72 h, a decreased proportion of pyknotic nuclei and a drastic reduction in the number of primordial follicles were indicative of a stable state after the CPA damage. Overall, the ovarian morphological profile in the control group remained stable throughout the 72 h of culturing, displaying dense primordial follicles in the cortex and some growing follicles within the medulla, contrasting with the CPA-treated ovaries with a sparse presence of primary follicles in the cortex by the end of the culturing. The quantification of the primordial follicle and growing follicle densities in both groups over the 72-h follow-up showed a sharp reduction in primordial follicles in the CPA-treated ovaries within the first 24 h (*p* < 0.05), followed by a less pronounced decline at 48 h (*p* < 0.0001), and a slower reduction at 72 h (*p* < 0.01) (**Figure 1B**). In contrast, the 4-HC treatment did not affect the density of the growing follicles, which were similar to the control group at all four time points.

**Figure 2:**
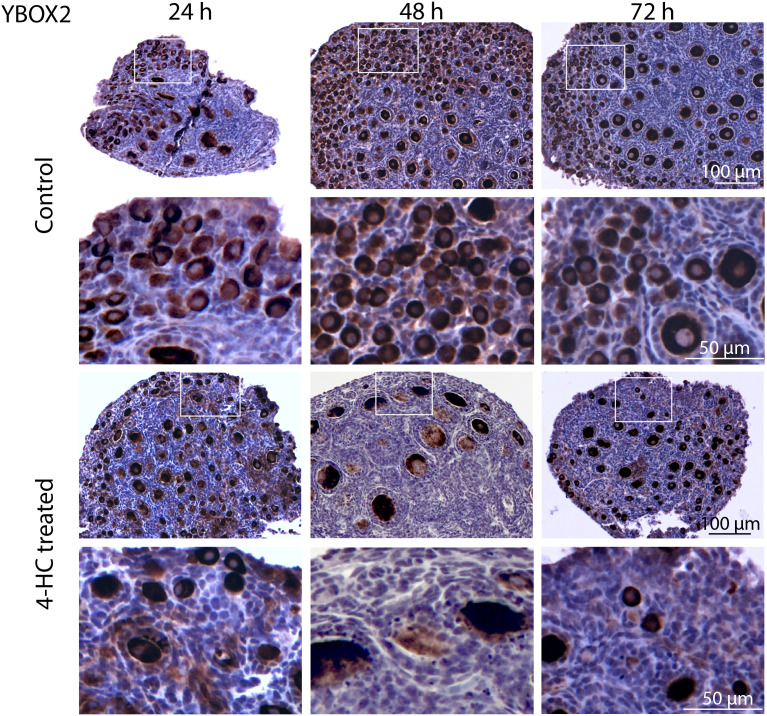
Immunohistochemistry staining of YBOX2 in control and 4-HC-treated ovaries over time. Brown-stained cells are YBOX2-positive. Each one involves an overview of the ovary and magnification of the cortex area marked by a square in the overview above.

**Figure 3:**
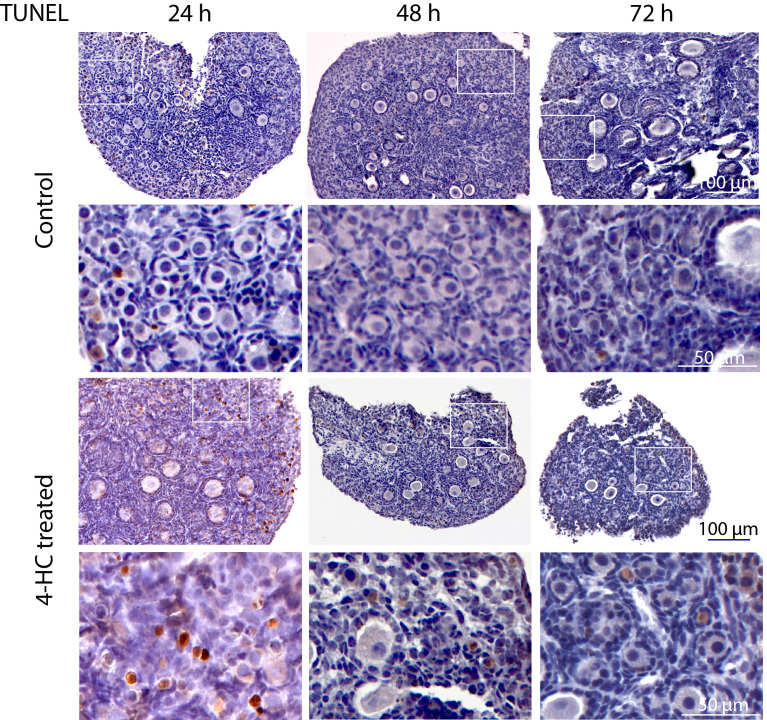
*In situ* detection of apoptosis-related DNA fragmentation by TUNEL assay in control and 4-HC-treated ovaries over time. Brown-stained cells are TUNEL-positive. Each one involves an overview of the ovary and the magnification of the cortex area marked by a square in the overview above.

To assess the distribution of the oocytes contained in the primordial and growing follicles, immunohistochemistry staining of YBOX2, a germ cell-specific nucleic acid-binding protein (46), was performed (**Figure 2**). The localization of YBOX2 positive cells was similar over the time points in the control ovaries, characterized by a higher density of YBOX2 positive cells along the cortex and sparse distributions in the medulla area. However, in 4-HC-treated group, the density of YBOX2 positive cells in the cortex area progressively decreased over time, while no obvious changes in YBOX2 positive cells across the medulla area were found. These results showed a correlation with the observed alterations in the ovarian profiles revealed by H&E staining (**Figure 1A**) and the dynamic primordial follicle and growing follicle density changes illustrated in **Figure 1B**. As shown in **Figure 3**, 4-HC-treated ovaries contained more TUNEL-positive cells at 24 h. These cells were mainly primordial follicle oocytes. At 48 and 72 h, the positive signals in 4-HC-treated ovaries decreased and were confined mainly to primordial follicle oocytes and few somatic cells, whereas the control ovaries scarcely showed TUNEL-positive cells throughout the 72-h culture.

**Figure 4:**
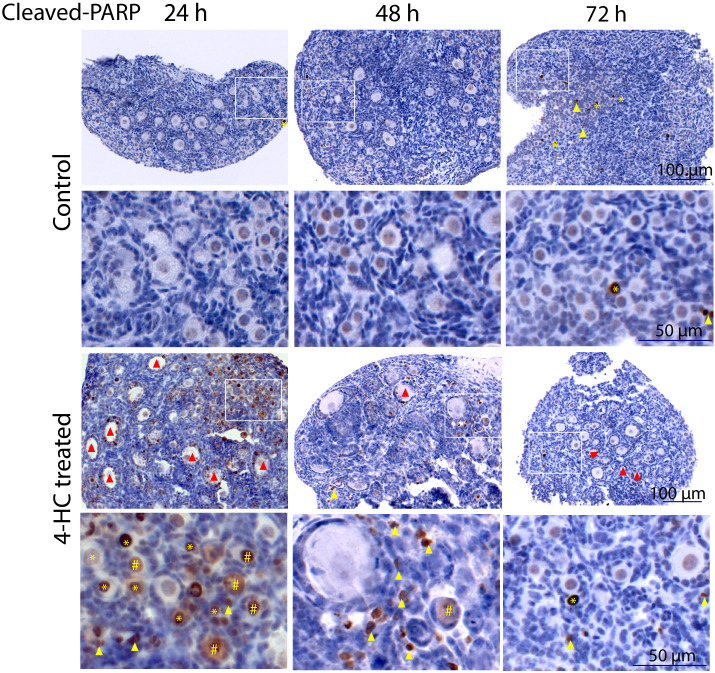
Immunohistochemistry staining of cleaved-PARP in control and 4-HC-treated ovaries over time. Brown-stained cells are cleaved-PARP-positive. Each one involves an overview of the ovary and the magnification of the cortex area marked by a square in the overview above. Marks: cleaved-PARP-positive primordial follicle oocytes (*), early growing follicle oocytes (#), cleaved-PARP-positive stromal cells (yellow triangles), growing follicles with cleaved-PARP-positive granulosa cells (red triangles).

**Figure 5:**
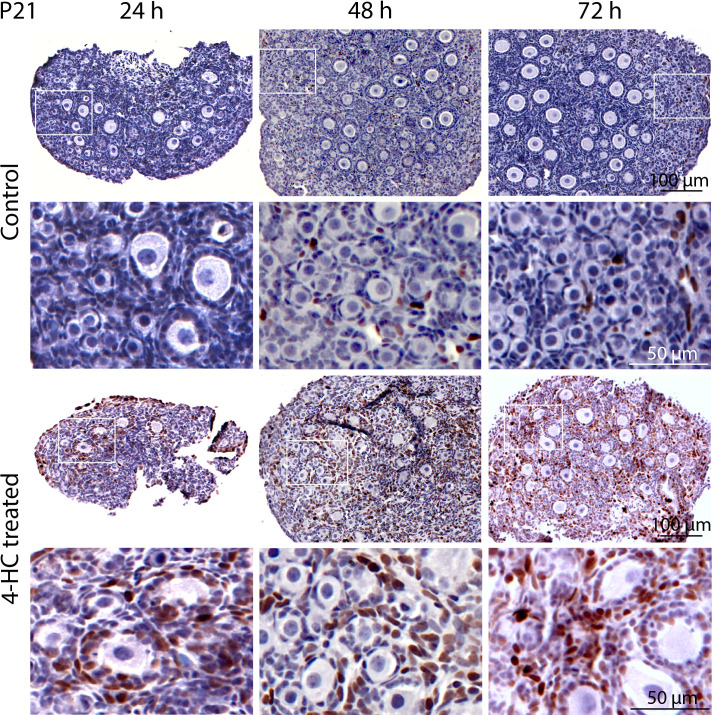
Immunohistochemical staining of p21 in control and 4-HC-treated ovaries over time. Brown-stained cells are p21-positive. Each one involves an overview of the ovary and the magnification of the cortex area marked by a square in the overview above.

**Figure 6:**
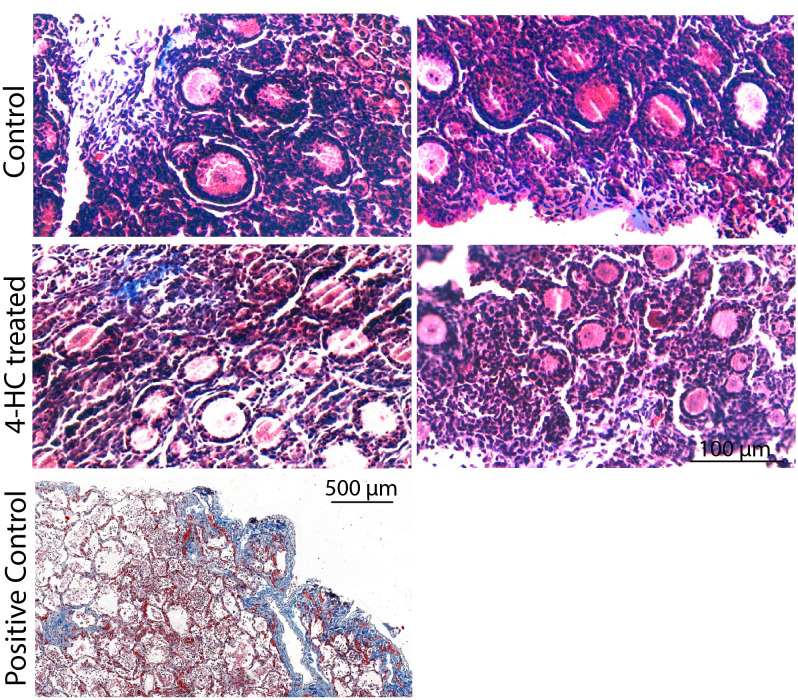
Masson’s staining in control and 4-HC-treated ovaries at 72 h. Masson Trichrome stains collagen fibers blue. Positive control: Human lung tissue.

Immunohistochemistry staining of cleaved-PARP was performed to track the downstream effect of 4-HC-introduced DNA damage. PARP is a ubiquitous DNA repair enzyme which can be cleaved by cleaved caspases into a catalytic fragment of 89 kDa and a DNA binding unit of 24 kDa upon apoptosis, and is thus usually used as a caspase-dependent apoptosis marker (47, 48). Similar to the dynamic profiles observed in the TUNEL assay, cleaved-PARP-positive cells were rarely seen in the control ovaries throughout the 72-h culture. However, in 4-HC-treated ovaries, several cleaved-PARP-positive cells were observed at 24 h (**Figure 4**). The locations of the cleaved-PARP positive cells were mainly in the oocytes of primordial follicles and early growing follicles, accompanied by the presence in some granulosa cells of growing follicles and ovarian stromal cells. At 48 h, cleaved-PARP-positive cells decreased in 4-HC-treated ovaries and were mainly observed in stromal cells. At 72 h, cleaved-PARP-positive cells were rarely observed in the 4-HC-treated ovaries at similar levels as the control ovaries. P21 is a member of the cyclin-dependent kinase (CDK) inhibitor family and plays multiple roles in response to DNA damage, apoptosis, and DNA repair (49). Immunohistochemistry staining of p21 in 4-HC-treated ovaries showed many p21 positive signals in stromal cells and granulosa cells of the growing follicles, especially at 48 and 72 h (**Figure 5**). In the control ovaries, p21 positive signals were rarely observed at 24 h, and at 48 and 72 h, a few p21 positive signals were detected, mainly in somatic cells. Masson’s staining was performed at 72 h considering that the impact on collagen composition could require a longer time, but the results (**Figure 6**) showed a similar small blue-stained area in both the control and 4-HC-treated ovaries.

**Figure 7:**
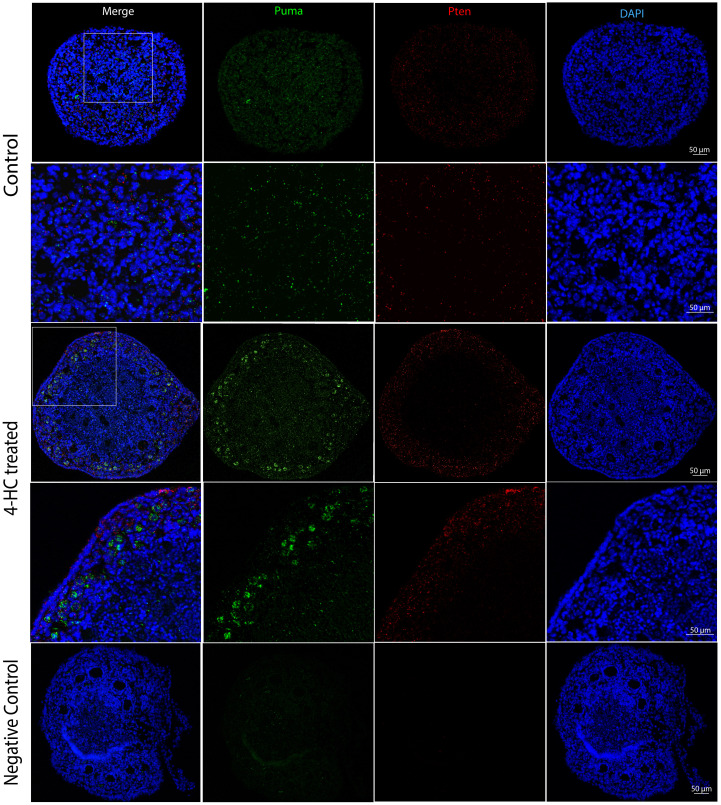
FISH-revealed localizations of *Puma* mRNA signals (Green) and *Pten* mRNA signals (red) in control and 4-HC-treated ovaries at 24 h, counterstained with DAPI (blue), and negative control. Images contain the whole section of the ovary and a magnification of selected areas containing the cortex and subcortical areas designated in the square in the overview above.

FISH was performed at 24 h when the most active apoptotic response to damage from CPA was observed (**Figure 7**). The distribution of *Puma* and *Pten* mRNA did not show a determined pattern in the control ovaries. By contrast, the 4-HC-treated ovaries showed increased signals of *Puma* in primordial follicles in the cortex area while *Pten* signals were scattered among stromal cells in the ovarian cortex.

### 3.2 Gene expression changes induced by CPA treatment in ovaries

To capture CPA-treatment-regulated expression changes among the genes related to potential signaling of CPA-induced ovarian damage, comprehensive gene expression profiling in the CPA-treated and control groups was performed dynamically. Eight groups of genes were targeted: 1) direct damage response to CPA: *H_2_ax*, *Sod1* and *p21*, also known as *Cdkn1a*; 2) apoptosis-related: *Casp 3*, *Casp 6*, *Casp 8*, *Trp53*, *Tap63*, *Noxa*, *Bax*, *Puma*, and *Bcl-2*; 3) primordial follicle survival maintenance-related: *Pdk1* and *Mtor*; 4) primordial follicle dormancy maintenance-related: *Pten* and *Foxo3a*; 5) Germ cell-specific gene *Ybx2;* 6) follicle development, angiogenesis, and neovascularization related: *Pcna*, *Mki67*, *Amh*, *Tgfb1*, *Smad2, Smad3*, and *Stat3*, *Cd31;* 7) Hippo pathway-related: *Yap1, Mst1*, and *Lats2*; 8) MAPK3/1 (mediates primordial follicle activation) signaling-related: *Kitl.*

**Figure 8:**
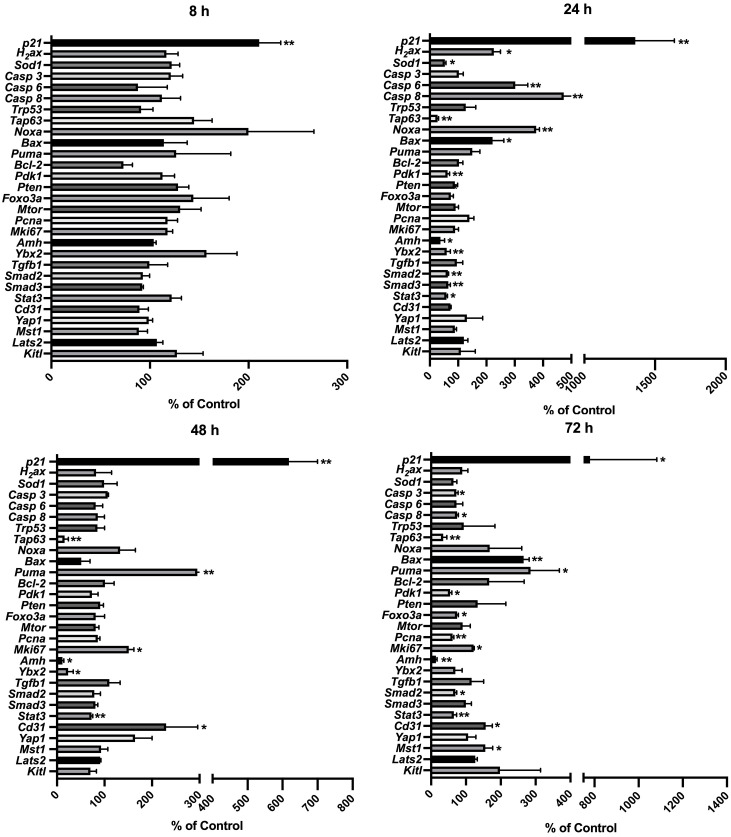
Relative expression levels of selected genes at different time points in the 4-HC-treated ovaries compared with the untreated control ovaries: ^*^ *p* < 0.05, ^**^ *p* < 0.01.

Among the analyzed genes (**Figure 8**)*, p21* showed the earliest response to 4-HC treatment; its expression increased up to 2.1 times compared to the control group at 8 h (*p* < 0.01). The expression of the other analyzed genes remained stable at 8 h after 4-HC exposure. At 24 h, *p21* showed the greatest increase in expression level of up to 13.6 times in the treated ovaries compared to the control group (*p* < 0.01). This significant increase continued to the end of the culture at 72 h, making it the only gene among all the tested genes that increased significantly at all time points. At 24 h, the DNA damage marker *H_2_ax* and the oxidative stress-related marker, ROS scavenger *Sod1*, were significantly upregulated and downregulated, respectively. Meanwhile, *Casp 6* and *8*, *Noxa*, and *Bax* were significantly upregulated. *Tap63*, *Pdk1*, *Ybx2*, and *Amh* were downregulated, indicating the loss of oocytes as well as damage in granulosa cells. Regarding TGFβ/SMAD signaling, which is relevant for follicular development, *Tgfb1* was not significantly regulated while *Smad2, Smad3*, and angiogenesis-related gene *Stat3* were downregulated. At 48 h, *Tap63*, *Ybx2*, *Amh*, and *Stat3* were continuously downregulated, while *Puma*, *Mki67*, and *Cd31* were significantly increased. At 72 h, *Casp 3* and *8* were slightly downregulated while *Bax* and *Puma* were upregulated; *Tap63*, *Pdk1*, *Amh, Smad2*, and *Stat3* were continuously downregulated; *Mki67, Cd31*, and *Mst1* were upregulated whereas *Pcna* was downregulated. Apoptosis inducer *Trp53*, apoptosis inhibitor *Bcl-2*, and *Mtor* did not show variations in gene expression over the entire time period. A regulator of primordial follicle dormancy, *Pten*, was not significantly altered at any time point, while another primordial follicle dormancy regulator, *Foxo3a*, only showed slight downregulation at 72 h.

### 3.3 Western blot analysis indicated CPA treatment increased the cleavage of PARP

**Figure 9:**
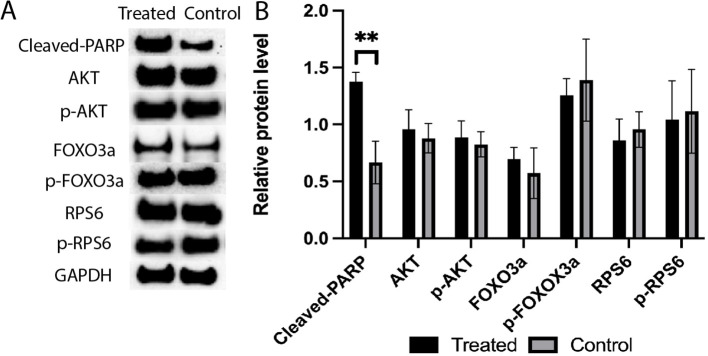
**(A)** Expression levels of cleaved-PARP, AKT, p-AKT, FOXO3a, p-FOXO3a, RPS6, and p-RPS6 proteins in the 4-HC-treated and control groups, detected by Western blotting. GAPDH was used as an internal control. **(B)** Protein expression levels were quantified using Image J, data mean ± SD, **P < 0.01.

Among the analyzed proteins (**Figure 9**), the cleavage of PARP was significantly increased in response to the 4-HC treatment compared to the control at 24 h (*p*<0.01). However, the relative levels of the other proteins analyzed, i.e., RPS6, FOXO3a, Akt, and their phosphorylated proteins, in the treated ovaries remained at similar levels as untreated controls.

## 4 Discussion

Herein, we offer a comprehensive overview of the early changes occurring in prepubertal mouse ovaries following *in vitro* exposure to 4-HC across several time points during the first 72 h. We provide new insights into the early events and underlying mechanisms driving primordial follicle depletion and active ovarian damage induced by CPA. Among our selected time points, we identified 24 h as the active period in which CPA actively damaged the ovaries and depleted the primordial follicle pool. Alterations induced by CPA were visualized through histological methods and complemented by gene expression analysis of the candidate genes and detection and quantification of key proteins to reflect CPA-triggered ovarian responses. Histological observations showed that the appearance of pyknotic nuclei in oocytes coincided with a significant reduction in primordial follicle density in the treated ovaries at 24 h. In our experiments, we did not find evidence of changes in growing follicle density throughout the 72 h following 4-HC treatment. Meanwhile, in the 4-HC-treated group, increased signals of DNA damage and apoptosis were observed using TUNEL assay and cleaved-PARP immunohistochemistry staining, and a significant increase in cleaved-PARP protein levels was detected by Western blot, as summarized in **Table 2**. Gene expression analysis showed a significant increase in *H_2_ax*, *Casp 6* and *8*, *Noxa*, and *Bax*, and significant downregulation of *Tap63*, *Pdk1*, *Ybx2*, and *Amh*, which suggests the involvement of apoptotic processes during ovarian follicle depletion, as summarized in **Table 2**. The visualization of fluorescent signals of upregulated *Puma* at 24 h provided further evidence of enhanced apoptosis in the primordial follicles. However, there were no obvious variations related to *Pten*. The continuous downregulation of *Pdk1* might result in the disruption of its role as a survival maintainer of dormant primordial follicles (50), its deficiency inducing rapid primordial follicle clearance directly from their quiescent status (50). Additionally, primordial follicle dormancy maintenance and activation, Hippo signaling-related key components, and cell proliferation-related genes were not altered by 4-HC treatment at 24 h (*Pten*, *Foxo3a, kitl, Yap1, Mst1, Lats2*, *Pcna* (51), and *Mki67*). Western blot analyses of the CPA-treated and control ovaries at 24 h did not show variations in the levels of phosphorylated proteins involved in primordial follicle activation, which are thought to specifically regulate this signaling pathway.

**Table 2:** Summary of the main changes in protein and gene expression levels caused by the 4-HC treatment at 24 h.

24 h	Overexpression ↑	Underexpression ↓
**Protein expression level**	P21 **↑↑** TUNEL-positive signals **↑** Cleaved-PARP **↑**	YBOX2 **↓**
**Gene expression Level**	*p21* **↑↑↑** *H_2_ax* **↑** *Casp 6, 8, Noxa, Bax, Puma ↑↑*	*Ybx2* **↓** *Sod1, Tap63, Pdk1, Amh* **↓** *Smad2, 3, Stat3* **↓**

One arrow indicates a low relative level of change, two arrows indicate a medium relative level of change, and three arrows indicate a high relative level of change.

Since we could not observe CPA-induced primordial follicle over-activation consistent with findings reported in previous studies (20, 24, 52), our results highlight apoptosis as the most likely driving mechanism in the early stages of CPA-induced primordial follicle depletion and ovarian damage. Analyzing the apoptosis results, we noticed a slight divergency of the TUNEL- and cleaved-PARP-positive signal localizations in the CPA-treated ovaries at 24 h. The cleaved-PARP signals were frequently observed in the primordial follicle oocytes, granulosa cells of the growing follicles, and some stromal cells, whereas the TUNEL-positive signals were mainly observed in the primordial follicle oocytes. This may suggest that somatic cells underwent cell death without clear evidence of DNA damage. This differential response could indicate a cell type-dependent susceptibility and distinct cell death triggers in response to CPA. Additionally, our results showed that *p21* mediated ovarian response to CPA treatment as early as 8 h, preceding the marked ovarian damage time point observed at 24 h. Immunohistochemical observation at 24 h showed a higher intensity of p21-positive signals in the granulosa cells of the growing follicles and also in stromal cells. Whilst other results showed strong primordial follicle damage caused by CPA, especially at 24 h, p21-positive signals were not observed in primordial follicle oocytes. P21 can promote cells to enter into cell cycle arrest and repair of DNA damage or into apoptosis when the DNA damage is unrepairable (53). Our findings suggest that CPA may cause different responses among different types of ovarian cells, which may correlate with the divergent distributions of TUNEL- and cleaved-PARP-positive cells reported herein. Another aspect concerns *Tap63*, which is expressed in the nucleus of primordial follicle oocytes and is regarded as a genomic safeguard of female germline cells, determining the fate of oocytes upon DNA damage (52, 54–56). A previous study identified *Tap63* as a mediator of apoptosis in oocyte death induced by CPA (54). Downregulation of *Tap63* started at 24 h and persisted at 48 and 72 h. This coincides with the drastic depletion in primordial follicle density that we observed at 24 h and which persisted until 72 h. Interestingly, previous studies have related p21 to both p53-dependent and p53-independent apoptotic signaling, transcription, cell differentiation, senescence, and DNA repair, especially in response to stimuli such as DNA damage (49, 57–59).

The damage induced during and after chemotherapy has been reported to extend throughout the ovarian micro-environment, influencing the immune microenvironment, vascular system, stromal cells, and extracellular matrix (60). In our study, we observed a downregulation of *Stat3*, which has been related to changes in the microvessel network (61). We identified a downregulation of *Smad2* and *Smad3* in TGFβ/SMAD signaling which are factors involved in neovascularization. However, it has been reported that these factors are also important for supporting ovarian follicle development (62, 63). The increase of *Cd31* at 48 and 72 h might be a compensatory response of ovaries to mediate the neovascularization damage from CPA. At the end of the 72-h follow-up, we also checked the potential impact of CPA on the fibrosis status in the ovaries. However, we did not observe significant changes, as similar patterns were observed in the treated and control ovaries. It is plausible that the duration of our observation may have been too short, and our culture setup has not been yet optimized for an extended culture that could provide deeper insights into this over a longer time.

Concerning the inferences about molecular biology that can be made from our study, we need to address several limitations, as we could only report on very early events within a 72-h time frame. There is also the possibility that alterations of genes and proteins related to primordial follicle activation, which concerns just a small number of entities such as oocytes and granulosa cells in the whole ovary, were not strong enough to be identified in the bulk RNA sequencing and protein homogenate of the whole organ. Future studies aiming to understand primordial follicle activation and underlying mechanisms could be conducted by integrating actual information on regulatory proteins into the topology of the follicle structure. In this regard, multi-omics assays designed as a time-course experiment such as ours could offer valuable and integrated information about different regulatory processes and, combined with spatial-omics, could also disentangle how they are integrated in the ovarian follicle. These new technologies might provide tools to better capture/target the changes involved in the primordial-to-primary follicle transition. Primordial follicle activation is a complex process that remains elusive, with approximately 1000 genes identified to be involved (64–66). In rodents, a complex intercellular signaling network driven by ligand and receptor interactions has been described in the primordial-to-primary follicle transition (67). In this study, we selected representative genes already known to be involved in these mechanisms. However, additional genes in this complex network were not investigated, nor were the effects in the mature ovaries of adult-age mice.

In conclusion, our study provides valuable insights into the early molecular mechanisms underlying CPA-induced ovarian damage in mice of a prepubertal age, emphasizing apoptosis and cellular responses within the ovarian follicle microenvironment as crucial in the early stages of primordial follicle depletion. We observed a significant impact as early as 24 h after the initial single exposure to CPA, highlighting the need for additional research to explore the broader implications of chemotherapy-induced ovarian injury and to develop protective therapies aimed at preventing such damage within this short time frame.

## Data Availability

The raw data supporting the conclusions of this article will be made available by the authors, without undue reservation.
